# Overview of infection control in nursing research in Korea over the last 10 years: Text network analysis and topic modeling

**DOI:** 10.1017/ash.2023.228

**Published:** 2023-09-29

**Authors:** EunJo Kim, JaHyun Kang

## Abstract

**Background:** With the emergence of new infectious diseases, infection control nursing (ICN) in hospitals has become increasingly significant. Consequently, research on ICN has been actively performed. We examined the knowledge structure and trends addressed in Korean ICN research. **Methods:** From 5 web-based Korean academic databases (DBpia, KISS, KMbase, KoreaMed, and RISS), 2,244 studies published between 2013 and 2022 were retrieved using ICN-related search terms (eg, “nurse” or “nursing” along with “infection control,” “infection prevention,” “healthcare-associated infection,” or “standard precautions”). After deleting duplicates, the authors assessed titles and abstracts and included 250 research abstracts in this study. Using NetMiner 4.4 software (Cyram, Seoul, Korea), words from abstracts of published articles were extracted and refined, then text network analysis and topic modeling were performed. A text network was structured based on the co-occurrence matrix of key words (semantic morphemes) and was analyzed to identify the main key words. Through topic modeling using the Latent Dirichlet Allocation algorithm, latent topics in the research abstracts were extracted. The authors verified the key words comprising the topic and the result of classifying the documents by topic and named topics. **Results:** The number of studies, which increased following the outbreak of Middle East respiratory syndrome in 2015, has declined over time but peaked in 2021 with the COVID-19 pandemic. The text network composed of the key words of the research abstracts was generated and visualized (Fig. 1). As a result of text network analysis, the 5 most common key words were ‘nurse,’ ‘infection control,’ ‘nursing care,’ ‘practice,’ and ‘perception’ in terms of degree and betweenness centrality. Other prominent main keywords were also identified: ‘knowledge,’ ‘compliance,’ ‘education,’ ‘intervention,’ ‘intention,’ and ‘safety.’ With the application of topic modeling to the research abstracts, 5 topics were derived and named as follows (Fig. 2): “infection control in nursing care for patient safety,” “infection control measures for healthcare personnel safety,” “burdens and obstacles for infection control among nurses,” “infection control for multidrug-resistant organisms,” and “knowledge, attitude, practice for infection control among nurses.” **Conclusions:** By applying text-network analysis and topic modeling, we obtained insights into Korean ICN research trends. To explore global ICN research trends, further study is necessary to analyze internationally published studies reflecting each country’s nursing work conditions.

**Figure:**
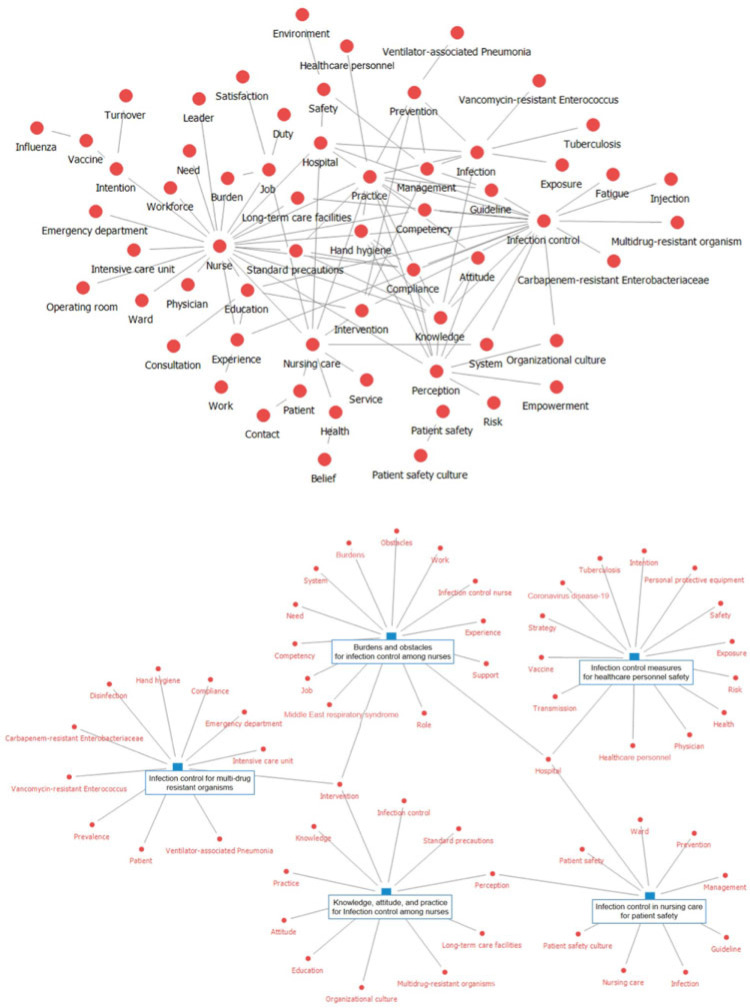

**Disclosure:** None

